# Co-infections with Multiple Viruses: A Frequent cause of Community-Acquired Pneumonia in Sarawak Malaysia

**DOI:** 10.1016/j.ijregi.2025.100748

**Published:** 2025-09-07

**Authors:** Teck-Hock Toh, Jeffrey Soon-Yit Lee, Sook-Min Yong, Nur Alfreena Binti Alfie, Siew-Ming Ting, Chew-Ee Wong, Kamilah Dahian, See-Chang Wong, Cheng-Foong Cheah, Anantha Raman Selvarajan, Bee-Shuang Lee, Judith U. Oguzie, Thang Nguyen-Tien, Claudia M. Trujillo-Vargas, Diego B. Silva, Emily R. Robie, Laura A. Pulscher, Mohd Raili Suhaili, Lyudmyla Marushchak, Gregory C. Gray

**Affiliations:** 1 Clinical Research Centre, Sibu Hospital, Ministry of Health Malaysia, Sibu, Malaysia; 2 Faculty of Medicine, Nursing & Health Sciences, SEGi University, Kota Damansara, Malaysia; 3 Department of Paediatrics, Sibu Hospital, Ministry of Health Malaysia, Sibu, Malaysia; 4 Department of Internal Medicine, Kapit Hospital, Ministry of Health Malaysia, Kapit, Malaysia; 5 Department of Anaesthesiology & Intensive Care, Sibu Hospital, Ministry of Health Malaysia, Sibu, Malaysia; 6 Department of Paediatrics, Sarikei Hospital, Ministry of Health Malaysia, Sarawak, Malaysia; 7 Department of Internal Medicine (Infectious Diseases), University of Texas Medical Branch, Galveston, USA; 8 Duke Global Health Institute, Duke University, Durham, USA; 9 Institute for Human Infections and Immunity, University of Texas Medical Branch, Galveston, USA; 10 Department of Microbiology and Immunology, University of Texas Medical Branch, Galveston, USA; 11 Department of Global Health, University of Texas Medical Branch, Galveston, USA

**Keywords:** Sarawak, Community-acquired pneumonia, Viral etiology, Molecular diagnostics, Epidemiology, Respiratory viruses

## Abstract

**Objectives:**

Equatorial Sarawak, Malaysia, has been the site of important novel respiratory virus detections. During the COVID-19 pandemic, we sought to determine viral causes of pneumonia that were not SARS-CoV-2.

**Methods:**

Using an informed consent process, we enrolled patients from four hospitals in Sarawak for this cross-sectional study. Patients permitted a nasopharyngeal (NP) swab collection and completed a risk factor questionnaire. We studied NP swabs with molecular diagnostics for previously recognized respiratory viruses such as influenza A and D viruses, and pan-species assays for adenoviruses, coronaviruses, enteroviruses, pneumoviruses, and paramyxoviruses.

**Results:**

Among 441 patients, 78.2% had at least one virus detected, and 24.9% had multiple viruses detected. Among the viruses detected, a commercial multiplexing assay found the most prevalent detections were human rhinoviruses (43.1%), respiratory syncytial virus (18.6%), human metapneumovirus (8.6%), influenza A (7%), adenovirus (6.1%), and influenza B (5.6%). However, the pan-species assays detected evidence of 19 additional respiratory viruses that the commercial multiplexing assay missed.

**Conclusions:**

Patients with pneumonia in this hot and humid region often had evidence of multiple viral infections, especially children under 5 years old. Clinicians who rely on singleplex molecular assays for prevalent viruses such as influenza A, SARS-CoV-2, and respiratory syncytial virus may miss other important viral causes of illness in such patients.

## Highlights

• We studied viral causes of pneumonia among hospitalized patients in Sarawak, Malaysia.
• 110 (24.9%) of the 441 patients had two or more viruses detected.
• Having previous contact with sick persons was a risk factor for viral coinfections.
• Relying upon a few singleplex molecular assays may mislead clinical staff.

## Graphical Abstract

**Figure:**
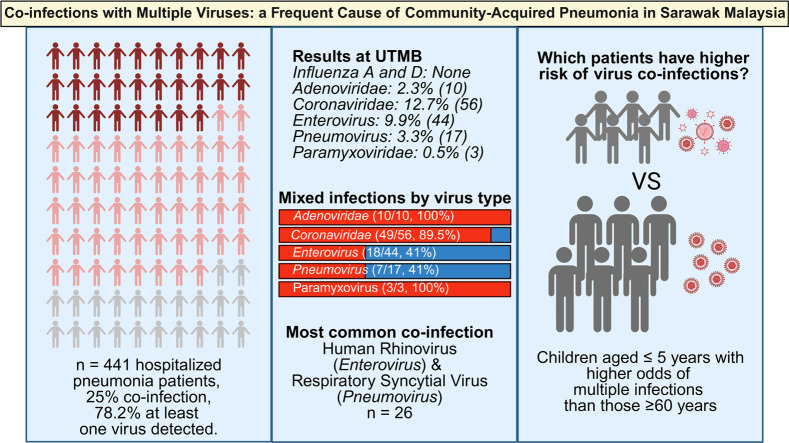

## Introduction

Despite the availability of numerous effective antimicrobials and vaccines, the world continues to suffer from emerging respiratory viruses. As evidenced by the recent H1N1 influenza A and SARS-CoV-2 pandemics in 2009 and 2020, emerging respiratory viruses cause much morbidity and mortality and are often difficult to control. The latter is partially due to their ability to spread rapidly and often sub-clinically, making the isolation of infected people nearly impossible [1]. Hence, respiratory viruses will continue to pose a frequent pandemic threat, and surveillance for novel strains is an important public health measure. One way to identify novel respiratory viruses is to study specimens from patients hospitalized with pneumonia in geographic regions known to be at risk for emerging respiratory threats. We have embraced such a strategy in our previous studies in northern Vietnam and East Malaysia [[2], [3], [4], [5]].

Here, we report results from a pneumonia etiology study in Sarawak, East Malaysia, where we have previously detected a novel canine-like coronavirus [4].

## Methods

### Study sites

The Malaysian state of Sarawak is located in East Malaysia. It comprises the northwestern part of the island of Borneo, the world’s third-largest island. Sarawak has a low-lying and heavily indented coastline along the South China Sea. Much of its area is covered by primary rainforest. The alluvial, swampy coastal plain is backed by rolling countryside that is intersected by mountains and numerous navigable rivers.

**Figure 1:**
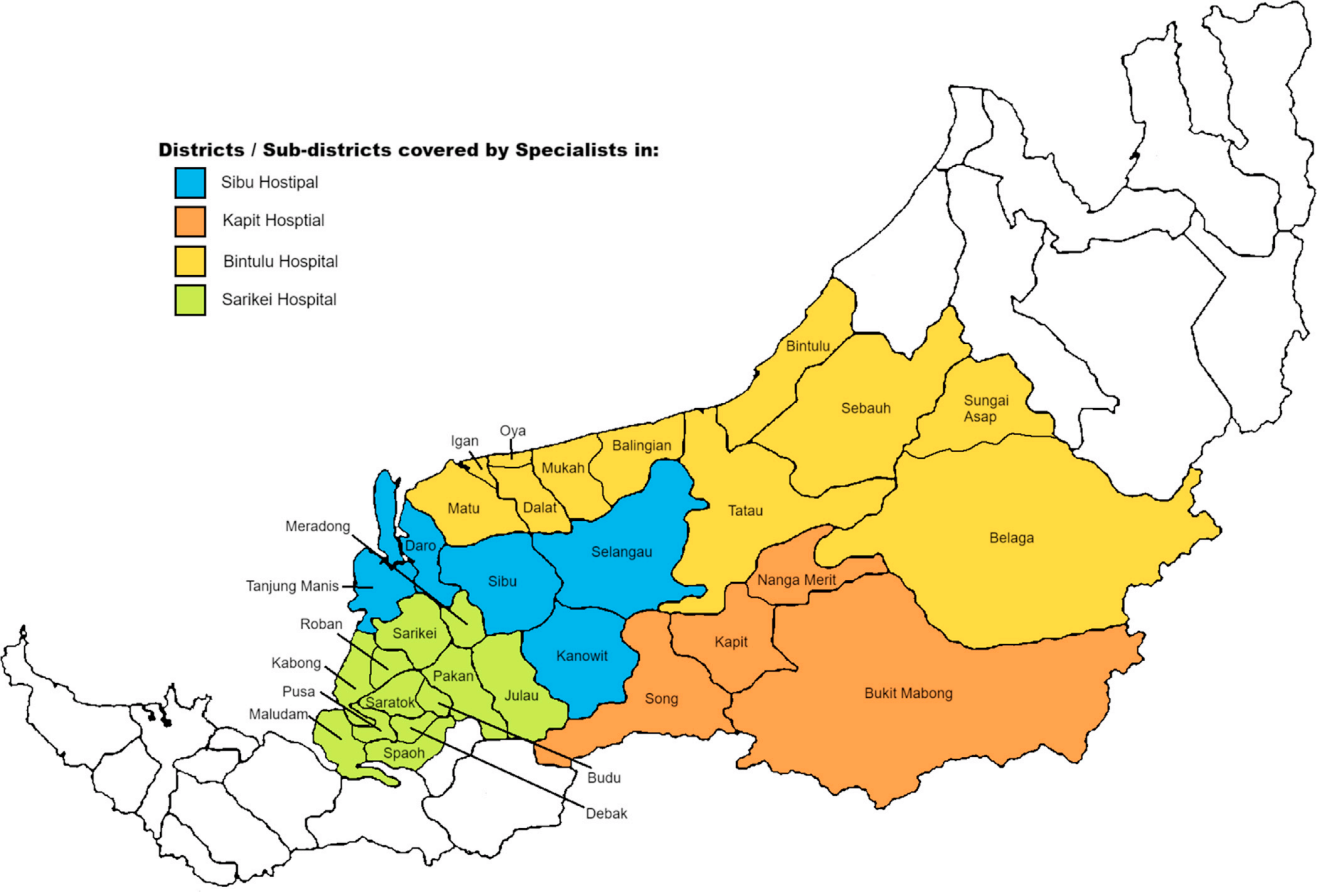
Patient catchment areas for the four hospitals in Sarawak, Malaysia where patients with pneumonia were enrolled. (Map generated using ChatGPT o4-mini-high for illustrative purposes).

This study was conducted at Sibu Hospital and three smaller district hospitals: Kapit Hospital, Bintulu Hospital, and Sarikei Hospital (Figure 1). Sibu Hospital is a regional and referral hospital for the central part of Sarawak, Malaysia. It has a bed capacity of 662 and serves as a referral hospital supporting six other smaller district hospitals that cater to a population of more than 725,400 people in the central region of Sarawak. The district hospitals of Kapit, Bintulu, and Sarikei have 134, 339, and 250 bed capacities, respectively.

### Study population

Patients of any age hospitalized in the four hospitals were eligible to participate if they met the following inclusion criteria: (i) had evidence of acute infection, defined as self-reported fever or chills with documented fever or hypothermia, leukocytosis or leukopenia, or new altered mental status less than seven days prior to admission; and (ii) had evidence of an acute lower respiratory illness, defined as new cough or sputum production, chest pain, dyspnea, tachypnea, abnormal lung examination, oxygen desaturation of 94% or less, or respiratory failure. Patients were excluded from participation if they met any of the following: (i) they had been hospitalized recently (<28 days for immunocompetent patients and <90 days for immunosuppressed patients); (ii) they had already enrolled in this study within the previous 28 days; (iii) they had a clear alternative diagnosis such as exacerbation of asthma or chronic obstructive airway disease, metabolic acidosis or heart disease to account solely for the respiratory symptoms; (iv) if they had structural abnormalities to the nasal passageway posting a risk of bleeding or trauma if nasopharyngeal (NP) swab was performed; or (v) tested positive for SARS-CoV-2 infection using a molecular assay or another test kit according to local practice guideline at time of hospital admission.

Study teams in Sarawak, Malaysia, employed informed consent when enrolling patients based on the inclusion and exclusion criteria. Eligible patients were briefed and given consent and enrollment questionnaire documents in the language of their choice (Chinese, English, or Malay). Study team members completed a questionnaire and collected an NP swab sample from the eligible patients. The questionnaire captured demographic information, health conditions, and animal exposures.

### Laboratory methods

NP swab specimens were placed in 3 mL of sterile viral transport media (Rocky Mountain Biologicals LLC), divided into three equal aliquots, and stored at -80 °C at Sibu Hospital’s Clinical Research Center Laboratory (CRC Lab): one aliquot was kept for local study, one aliquot was shipped to the University of Texas Medical Branch (UTMB) for further study, and one aliquot was held at the local site for back up purposes. Swab specimens were initially studied at Sibu Hospital’s CRC Lab with a commercial kit (Allplex^TM^ RV Essential Assay Seegene Inc., Seoul, South Korea) for influenza A (IAV), influenza B (IBV), adenovirus (AdV), human metapneumovirus (HMPV), human rhinoviruses (HRV), respiratory syncytial virus A/B/C (RSV), and parainfluenza virus (PIV).

At UTMB’s One Health Laboratory, the NP swab specimens were further studied using molecular assays for influenza D (IDV) and pan-species assays for adenoviruses, coronaviruses, enteroviruses, pneumoviruses, and paramyxoviruses as per our previously reported pan-species assay methods [6]. Pan-species assays that yielded amplicons of expected molecular weight were further studied with Sanger sequencing by Azenta Life Sciences (https://www.azenta.com). The resulting sequence data were studied using Geneious Prime® version 2025.1.2 for sequence editing and alignment. Sequences were then compared against the National Center for Biotechnology Information (NCBI) nucleotide database using the Nucleotide Basic Local Alignment Search Tool (BLASTn) to determine species identity based on the highest sequence similarity.

### Data analysis

Questionnaire data were entered into Microsoft Excel for data management and then imported into R software version 4.1.1 for analysis. Counts and percentages were used for descriptive statistical analyses.

Single infection was defined as a specimen with only one positive result from commercial and molecular tests performed by Sibu Hospital’s CRC Lab and/or UTMB’s One Health Laboratory. Coinfection was defined as a specimen with positive results for at least two or more viruses from commercial and molecular tests performed by either laboratory. Having any viral infection was defined as a specimen with positive results from any tests performed by either laboratory.

We modeled patients with evidence of any viral infection and multiple viral infections, considering assays run at both Sibu Hospital’s CRC and UTMB’s One Health laboratories. We first examined bivariate risk factor associations with Chi-square or Fisher’s exact tests, calculating odds ratios (ORs) with 95% confidence intervals (CIs). Potential risk factors with a bivariate test statistic *P* < 0.1 were included in stepwise, automatic, backward-elimination, unconditional logistic regression models. Risk factors with *P* < 0.05 were retained in the final logistic regression models.

## Results

From April 2022 to March 2023, study teams in Malaysia enrolled a total of 441 patients with pneumonia. A total of 256 patients (58.1%) were from Sibu Hospital, 99 (22.4%) from Kapit Hospital, 61 (13.8%) from Sarikei Hospital, and 25 (5.7%) from Bintulu Hospital (Table 1). In total, 181 patients (41.2%) were female. Children under five years old accounted for the majority of patients (n = 249, 56.7%). Most of the patients’ ethnicities were Iban (n = 269, 63.0%), followed by Malay (n = 81, 19.0%). Almost half of patients (n = 204, 46.3%) did not have any chronic illnesses, while 34.2% (n = 151) confirmed that they had at least one chronic illness. Relatively few patients (n = 53, 12%) reported current smoking or previous smoking. A quarter of patients (n = 111, 25.2%) reported contact with sick people before hospitalization. More than half (n = 238, 54%) of the patients reported no recent close contact with animals. Only 4.1% (n = 18) of patients traveled outside the study sites of Sarawak (Table 1).

**Table 1:** Characteristics of patients hospitalized with pneumonia in Sarawak, Malaysia, enrolled in the study from April 2022 to March 2023.

Demographic characteristics	Sibu Hospital n = 256 n (%)	Kapit Hospital n = 99 n (%)	Sarikei Hospital n = 61 n (%)	Bintulu Hospital n = 25 n (%)	Total n = 441 n (%)
**Gender**					
Male	154 (60.4)	52 (53.1)	37 (60.6)	15 (60.0)	258 (58.8)
Female	101 (39.6)	46 (46.9)	24 (39.4)	10 (40.0)	181 (41.2)
**Age**					
0-5 years	164 (64.6)	15 (15.1)	47 (77.0)	23 (92.0)	249 (56.7)
6-17 years	25 (9.8)	9 (9.1)	9 (14.8)	2 (8.0)	45 (10.3)
18-59 years	40 (15.8)	33 (33.3)	3 (4.9)	0	76 (17.3)
≥60 years	25 (9.8)	42 (42.4)	2 (3.3)	0	69 (15.7)
**Ethnicity**					
Iban	146 (57.3)	83 (90.2)	32 (58.2)	8 (32.0)	269 (63.0)
Malay	52 (20.4)	3 (3.3)	20 (36.4)	6 (24.0)	81 (19.0)
Chinese	26 (10.2)	3 (3.3)	3 (3.3)	0	32 (7.5)
Melanau	24 (9.4)	0	0	4 (16.0)	28 (6.5)
Others	7 (2.7)	3 (3.3)	0	7 (28.0)	17 (4.0)
**Chronic illness**					
Yes	97 (37.9)	52 (52.6)	0	2 (8.0)	151 (34.2)
No	157 (61.3)	23 (23.2)	2 (3.3)	22 (88.0)	204 (46.3)
Unknown	2 (0.8)	24 (24.2)	59 (96.7)	1 (4.0)	86 (19.5)
**Smoking status**					
Currently	17 (6.6)	5 (5.0)	0	0	22 (5.0)
Prior	14 (5.5)	17 (17.2)	0	0	31 (7.0)
Never	223 (87.1)	66 (66.7)	2 (3.3)	25 (100.0)	316 (71.7)
Unknown	2 (0.8)	11 (11.1)	59 (96.7)	0	72 (16.3)
**Close contact with live animals**					
Yes	105 (41.0)	9 (9.1)	1 (1.65)	3 (12.0)	118 (26.7)
No	148 (57.8)	67 (67.7)	1 (1.65)	22 (88.0)	238 (54.0)
Unknown	3 (1.2)	23 (23.2)	59 (96.7)	0	85 (19.3)
**Sick contact**					
Yes	92 (35.9)	10 (10.1)	0	9 (36.0)	111 (25.2)
No	139 (54.3)	63 (63.6)	0	11 (44.0)	213 (48.3)
Unknown	25 (9.8)	26 (26.3)	61 (100.0)	5 (20.0)	117 (26.5)
**Travel history outside Sarawak**					
Yes	15 (5.9)	3 (3.0)	0	0	18 (4.1)
No	239 (93.3)	73 (73.8)	2 (3.3)	25 (100.0)	339 (76.9)
Unknown	2 (0.8)	23 (23.2)	59 (96.7)	0	84 (19.0)
**Single virus infection**					
Yes	139 (54.3)	45 (45.4)	35 (57.4)	16 (64.0)	235 (53.3)
No	117 (45.7)	54 (54.6)	26 (42.6)	9 (36.0)	206 (46.7)
**Multiple virus infection**					
Yes	74 (28.9)	10 (10.1)	19 (31.1)	7 (28.0)	110 (24.9)
No	182 (71.1)	89 (89.9)	42 (68.9)	18 (72.0)	331 (75.1)
**Any viral infection**					
Yes	213 (83.2)	55 (55.5)	54 (88.5)	23 (92.0)	345 (78.2)
No	43 (16.8)	44 (44.5)	7 (11.5)	2 (8.0)	96 (21.8)

Among 441 patients, 345 (78.2%) had at least one virus detected (Table 1), with 110 (31.9%) of these 345 having two or more viruses identified. The Allplex^TM^ RV Essential Assay results (Table 2) identified evidence of various viruses present in patients’ NP swabs, with the highest percentage for HRV (n = 190, 43.1%), followed by RSV (n = 82, 18.6%), HMPV (n = 38, 8.6%), IAV (n = 31, 7%), AdV (n = 27, 6.1%), IBV (n = 25, 5.6%), and PIV (n = 18, 4.1%). Among the positive samples of each virus, the proportion of single viral infection for IAV was 77.4% (n = 24), HRV (n = 123, 64.7%), HMPV (n = 18, 47.4%), RSV (n = 35, 42.7%), IBV (n = 11, 44%), PIV (n = 5, 27.8%), and AdV (n = 3, 11.1%). Molecular assays at UTMB identified that 12.7% (n = 56) of 441 specimens were positive by our pan-coronavirus assay, 9.9% (n = 44) were positive by our pan-enterovirus assay, 3.8% (n = 17) were positive by our pan-pneumovirus assay, and 2.3% (n = 10) were positive by our pan-adenovirus assay. Only three samples (0.7%) had molecular evidence of a paramyxovirus. None of the NP swabs had molecular evidence of IDV and CCoV-HuPn-2018 (Table 2). Numerous patients had evidence of multiple viruses. Of the coinfected specimens, the most common combination was HRV and RSV (n = 26).

**Table 2:** Molecular assay results from Sibu Hospital Clinical Research Center Laboratory and the UTMB One Health Laboratory.

Viral assays	Specimens with any viral detections n (%)	Single virus detected n (%)	More than one virus detected n (%)
**Sibu Hospital CRC Laboratory assays (n = 441)**			
Adenovirus	27 (6.1%)	3 (11.1%)	24 (88.9%)
Influenza A virus	31 (7.0%)	24 (77.4%)	7 (22.6%)
Influenza B virus	25 (5.6%)	11 (44.0%)	14 (56.0%)
Metapneumovirus	38 (8.6%)	18 (47.4%)	20 (52.6%)
Parainfluenza virus	18 (4.1%)	5 (27.8%)	13 (72.2%)
Respiratory syncytial virus	82 (18.6%)	35 (42.7%)	47 (57.3%)
Human rhinovirus A/B/C	190 (43.1%)	123 (64.7%)	67 (35.3%)
**UTMB One Health Laboratory assays (n = 441)**			
Influenza D virus	0	0	0
Pan-coronavirus	56 (12.7%)	7 (12.5%)	49 (87.5%)
Pan-adenovirus	10 (2.3%)	0 (0%)	10 (100%)
Pan-enterovirus	44 (9.8%)	26 (59.1%)	18 (40.9%)
Pan-pneumovirus	17 (3.8%)	10 (58.8%)	7 (41.2%)
Pan-paramyxovirus	3 (0.7%)	0 (0%)	3 (100%)
CCoV-HuPn-2018	0	0	0

In 56 specimens that were positive by the pan-coronavirus assay (Supplemental Table 1), Sanger sequencing indicated four had evidences of SARS-CoV-2, three had evidences of the human coronavirus OC43 strain, and the remaining specimens yielded no specific strain.

Ten samples examined with the pan-adenovirus assay yielded interpretable results by Sanger sequencing (Supplemental Table 1): eight were identified as human adenovirus 1, 2, 5, or 6, and two were identified as more closely aligned with mastadenovirus C.

Among 44 NP swabs that were positive by the pan-enterovirus assay, Sanger sequencing revealed multiple subtypes (Supplemental Table 1): Eleven samples were identified as Enterovirus D68 (EV-D68). Coxsackievirus A was detected in three samples, including types A4 and A10. We identified 10 distinct HRV A types: A1, A9, A15, A22, A29, A51, A55, A64, A81, and unclassified HRV-A strains (n = 6). Nine samples were positive for HRV C, including HRV-C21, C26, C32, C53, and C5. HRV B52 was detected in a single sample.

Among 17 samples that were positive for pan-pneumovirus sequencing data revealed that 93.8% were RSV A. Only one sample was characterized as HMPV.

Overall, UTMB’s pan-species laboratory work detected evidence of 14 enteroviruses, two each of pneumoviruses and paramixoviruses, and one AdV that the commercial multiplexing assay missed.

We conducted stepwise logistic regression for the outcomes of getting any viral infection and multiple viral infections (Table 3). Adults aged 18-59 years old, children aged 6-17 years old, and children ≤5 years old had 2.37 times (95% CI: 1.18, 4.80), 13.37 times (95% CI: 4.65, 48.91), and 10.79 times (95% CI: 5.64, 21.21) higher odds of having evidence of any viral infection compared to the elderly ≥60 years old. Malay people also had 3.03 times (95% CI: 1.28, 8.38) higher odds of getting any viral infection than Iban people. Children aged ≤5 years had significantly higher odds of having multiple infections than those ≥60 years (OR = 4.38, 95% CI: 2.03, 10.93). Patients who had contact with someone recently sick were at higher odds of having multiple infections compared to those without such exposure (OR = 1.85, 95% CI: 1.13, 3.03).

**Table 3:** Bivariate and multivariable logistic regression models of risk factors for viral infection among the patients with pneumonia.

Risk factors	Any viral infection			Multiple infections		
	Total n (%)	Bivariate ORs (95% CI)	Adjusted ORs (95% CI)	Total n (%)	Bivariate ORs (95% CI)	**Adjusted ORs (95% CI)**
**Age groups**						
0-5 years	225 (65.4)	12.2 (6.5, 23.4)	10.8 (5.6, 21.2)	88 (80.7)	4.8 (2.3, 12.0)	4.4 (2.0, 10.9)
6-17 years	41 (11.9)	13.3 (4.7, 48.0)	13.4 (4.7, 48.9)	11 (10.1)	2.9 (1.0, 8.4)	2.6 (0.9, 7.7)
18-59 years	48 (13.9)	2.2 (1.2, 4.4)	2.4 (1.2, 4.8)	3 (2.8)	0.4 (0.08, 1.4)	0.4 (0.1. 1.3)
>60 years	30 (8.8)	Reference	Reference	7 (6.4)	Reference	Reference
**Hospitals**						
Kapit	55 (15.9)	0.3 (0.2, 0.4)	—-	10 (9.1)	0.28 (0.1, 0.5)	—-
Sarikei	54 (15.7)	1.6 (0.7, 4.0)	—-	19 (17.3)	1.11 (0.6, 2.0)	—-
Bintulu	23 (6.7)	2.3 (0.7, 14.8)	—-	7 (6.4)	0.9 (0.4, 2.3)	—-
Sibu	213 (61.7)	Reference	—-	74 (67.2)	Reference	—-
**Ethnic groups**						
Malay	75 (22.4)	4.5 (2.0, 11.9)	3.0 (1.3, 8.4)	27 (24.8)	—-	—-
Melanau	26 (7.7)	4.7 (1.3, 29.4)	3.8 (1.0, 25.3)	9 (8.2)	—-	—-
Chinese	23 (6.9)	0.9 (0.4, 2.2)	1.2 (0.5, 3.0)	6 (5.5)	—-	—-
Others	13 (3.9)	1.3 (0.4, 4.2)	0.8 (0.2, 3.4)	6 (5.5)	—-	—-
Iban	198 (59.1)	Reference	Reference	61 (56.0)	—-	—-
**Chronic illness**						
Yes	97 (28.1)	0.3 (0.2, 0.5)	—-	19 (17.3)	0.3 (0.2, 0.5)	—-
No/Unknown	248 (71.2)	Reference	—-	91 (82.7)	Reference	—-
**Smoking status**						
Prior	17 (4.9)	1.0 (0.3, 3.2)	—-	2 (1.8)	0.7 (0.1, 6.1)	—-
Never	258 (74.8)	3.7 (1.5, 9.0)	—-	87 (79.1)	3.8 (1.2, 24.1)	—-
Unknown	58 (16.8)	3.4 (1.2, 9.7)	—-	19 (17.3)	3.6 (0.9, 23.8)	—-
Currently	12 (3.5)	Reference	—-	2 (1.8)	Reference	—-
**Sick contact**						
Yes	97 (28.1)	2.29 (1.27, 4.39)	—-	41 (37.3)	2.21 (1.38, 3.53)	1.85 (1.13, 3.03)
No/Unknown	248 (71.2)	Reference	—-	69 (62.7)	Reference	Reference

CI = confidence interval; ORs = odd ratios.

## Discussion

Our study found a high prevalence of viral infections among patients with pneumonia admitted to four hospitals in the Sarawak region of Malaysia. These results were similar to a study conducted in a tertiary hospital in West Malaysia, where 67.9% of patients with acute lower respiratory tract infections had evidence of infection with at least a single virus and 27.9% had evidence of multiple viruses [7]. However, that study differed in that the authors only studied 165 children under the age of five. Our viral detection prevalence (78.2%) was remarkably higher than the previous studies in the same Southeast Asia region [[8], [9], [10]]. The coinfection prevalence (24.9%) was also higher than in other pneumonia etiology studies in Singapore, Vietnam, the United Kingdom (UK), and the U.S. [[9], [10], [11], [12], [13], [14]], where coinfection was detected among 3% to 18% of patients. Nevertheless, our coinfection prevalence was lower than previous findings among 279 children (78%) at a tertiary-care university hospital in Brazil [15]. The differences could be explained by variations in sample collection time, sample size, study subjects, and diagnostic methods used in each study.

Detections of HRV and RSV were most prevalent in our study, consistent with other reports that HRV and RSV are the most prevalent viral pathogens among children with acute respiratory illness or community-acquired pneumonia [[14], [15], [16], [17], [18]]. The substantial detection rate of HRV (43.1%) warrants attention, as its clinical significance is often underappreciated due to perceptions of it causing only mild disease. HRV and RSV coinfection was the most common coinfection in our study, consistent with previous studies in France, Taiwan, the UK, and Vietnam [14,[19], [20], [21]]. Notably, we detected several complex coinfections involving viruses from up to four different viral families. This aligns with another study [13], reflecting high viral circulation and transmission intensity in this population. The public health significance of such multiple viral infections is not well understood, but some reports suggest that these infections are associated with increased disease severity [12], or even an increased mortality risk [22].

Additionally, our results reveal a relatively high percentage of patients with evidence of coronavirus (12.7%). Sanger sequencing confirmed SARS-CoV-2 in four patients’ swabs even though our inclusion criteria excluded the patients with positive results for SARS-CoV-2 infection at the time of hospital admission. Since the sample collection was conducted in the context of the ongoing COVID-19 pandemic, we are unsure if the detections were missed by the hospital assays or the viruses were acquired nosocomially.

Previously, we identified a novel canine coronavirus, referred to as CCoV-HuPn-2018, in a patient’s swab from Sarawak [4]. However, in this current study, we did not find evidence of CCoV-HuPn-2018, which might be due to the low prevalence or seasonal variation of this pathogen.

In our study, patients under five years of age were among the most vulnerable groups infected with viruses. Specifically, they had significantly higher odds of having any viral infection compared to patients ≥60 years old. They also had significantly higher odds of having multiple viral infections compared to patients aged ≥60 years old. These findings were consistent with a systematic review [23] and other U.S. studies suggesting that children often experience a significantly higher prevalence of coinfections than other groups [11,24,25].

Our findings of a high prevalence of viral infections may reflect a combination of age-related immunity, differential exposure, or care-seeking behavior. However, the findings also suggest that younger children remain a critical target for prevention efforts such as vaccination and public health messaging [8]. It was interesting to observe (Table 1) that multiple viral infections were more prevalent at Sibu Hospital, which is located in a geographic area with a higher population density. However, before one can attribute multiple viral infections to denser populations, we must also recognize Sibu Hospital as a referral hospital where the most seriously ill patients are often referred. The high prevalence of such viral infections in the hot and humid equatorial region of Sarawak is perhaps counterintuitive to models suggesting higher viral transmission in closed buildings in colder climates.

Importantly, patients who had contact with other sick individuals had 85% higher odds of having multiple viral infections compared to those who did not have contact with sick people at home or the workplace. This finding emphasizes the potential role of human-to-human transmission of multiple respiratory viruses and supports the need for viral diagnostics, especially in high-transmission settings such as households, schools, and clinics.

To our knowledge, this is one of the most comprehensive molecular surveillance efforts for respiratory viruses in this region, incorporating both commercial multiplex assays and in-house, pan-species conventional reverse transcription-polymerase chain reaction assays with sequencing confirmation. The pan-species methods enhanced viral detection; however, both approaches have intrinsic limitations. The commercial assays are constrained by fixed target panels and may miss novel or unexpected pathogens. On the other hand, pan-species assays require advanced laboratory infrastructure, extended specimen processing times, and specialized expertise for sequencing and interpretation. These factors may limit their feasibility for routine use in resource-limited settings such as Sarawak. Future studies should explore the cost-effectiveness and clinical utility of integrating such broad-spectrum diagnostics into standard care.

We acknowledge that our study was not without limitations. We did not capture data regarding many potential bacterial causes of pneumonia, and such data had great potential as confounding causes of morbidity. The quality of data and maintenance of NP specimens at -80°C differed between hospitals, with Sibu Hospital performing better than the more rural hospitals.

Even with these limitations, our findings demonstrate a high prevalence of both single and multiple respiratory viral infections among patients hospitalized for pneumonia in Sarawak. As molecular diagnostics for respiratory viruses are not uniformly available in Sarawak, these data support their need, especially for multiplexing diagnostics, which can detect multiple pathogens. This will be especially true as more specific therapies and vaccines against respiratory viral infections become available to healthcare providers in Sarawak.

## Declaration of competing interest

The authors have no competing interests to declare.

## Appendix Supplementary materials

Image, application 1
