# Supplementary material for: Co-infections with Multiple Viruses: A Frequent cause of Community-Acquired Pneumonia in Sarawak Malaysia
Source: IJID Reg. 2025 Sep 7;17:100748. doi: 10.1016/j.ijregi.2025.100748 (PMC12538036; doi:10.1016/j.ijregi.2025.100748)
Supplement: Supplementary file 1 [file mmc1.docx]

**Supplemental Information**

**Co-infections with Multiple Viruses: a Frequent cause of Community Acquired Pneumonia in Sarawak Malaysia**

Teck-Hock Toh,^1-3^ Jeffrey Soon-Yit Lee,^1^ Sook-Min Yong,^1^ Nur Alfreena Binti Alfie,^1^ Siew-Ming Ting,^1^ Chew-Ee Wong,^2^ Kamilah Dahian,^1^ See-Chang Wong,^2,3^ Cheng-Foong Cheah,^4^ Anantha Raman Selvarajan,^5^ Bee-Shuang Lee,^6^ Judith U. Oguzie,^7^ Thang Nguyen-Tien,^7^ Claudia M. Trujillo-Vargas,^7^ Diego B. Silva,^7^ Emily R. Robie,^8^ Laura A. Pulscher,^7^ Mohd Raili Suhaili,^2^ Lyudmyla Marushchak,^7^ and Gregory C. Gray^7,9-11^

^1^ Clinical Research Centre, Sibu Hospital, Ministry of Health Malaysia, Sibu, Sarawak, Malaysia

^2^ Faculty of Medicine, Nursing & Health Sciences, SEGi University, Kota Damansara, Selangor, Malaysia

^3^ Department of Paediatrics, Sibu Hospital, Ministry of Health Malaysia, Sibu, Sarawak, Malaysia

^4^ Department of Internal Medicine, Kapit Hospital, Ministry of Health Malaysia, Kapit, Sarawak, Malaysia

^5^ Department of Anesthesiology & Intensive Care, Sibu Hospital, Ministry of Health Malaysia, Sibu, Sarawak, Malaysia

^6^ Department of Pediatrics, Sarikei Hospital, Ministry of Health Malaysia, Sarikei, Sarawak, Malaysia

^7^ Department of Internal Medicine (Infectious Diseases), University of Texas Medical Branch, Galveston, Texas, USA

^8^ Duke Global Health Institute, Duke University, Durham, North Carolina, USA

^9^ Institute for Human Infections and Immunity, University of Texas Medical Branch, Galveston, TX, USA.

^10^ Department of Microbiology and Immunology, University of Texas Medical Branch, Galveston, TX, USA.

^11^ Department of Global Health, University of Texas Medical Branch, Galveston, TX, USA.

| **Supplemental Table 1.** Nasopharyngeal swab specimens that permitted virus characterization via pan-species molecular diagnostics followed by good data of Sanger sequencing. | | | |
| --- | --- | --- | --- |
| **Specimen ID** | **Identified Virus** | **Type** | **Accession number** |
| SBP-063 | Enterovirus | Coxsackievirus A10 | PV776105 |
| SAP-052 | Enterovirus | Rhinovirus A22 | PV776106 |
| SAP-045 | Enterovirus | Coxsackievirus A4 | PV776107 |
| SBP118 | Enterovirus | Coxsackievirus A4 | PV776108 |
| ICU-041 | Enterovirus | Rhinovirus A51 | PV776109 |
| SAP-025 | Enterovirus | Rhinovirus A9 | PV776110 |
| BTP-020 | Enterovirus | Rhinovirus B52 | PV776111 |
| ICU-035 | Enterovirus | Rhinovirus C21 | PV776112 |
| SBP-170 | Enterovirus | Rhinovirus C26 | PV776113 |
| SBP-160 | Enterovirus | Rhinovirus C26 | PV776114 |
| SBP-186 | Enterovirus | Rhinovirus C26 | PV776115 |
| SBP-185 | Enterovirus | Rhinovirus C53 | PV776116 |
| SAP-006 | Enterovirus | Enterovirus D68 | PV776117 |
| SAP-011 | Enterovirus | Enterovirus D68 | PV776118 |
| SAP-027 | Enterovirus | Enterovirus D68 | PV776119 |
| SBP-025 | Enterovirus | Enterovirus D68 | PV776120 |
| SBP-038 | Enterovirus | Enterovirus D68 | PV776121 |
| SBP-045 | Enterovirus | Enterovirus D68 | PV776122 |
| SBP-054 | Enterovirus | Enterovirus D68 | PV776123 |
| SBP-064 | Enterovirus | Enterovirus D68 | PV776124 |
| SBP-067 | Enterovirus | Enterovirus D68 | PV776125 |
| SBP-109 | Enterovirus | Enterovirus D68 | PV776126 |
| SBP-117 | Enterovirus | Enterovirus D68 | PV776127 |
| KPP-002 | Enterovirus | Rhinovirus A1B | PV776128 |
| SAP-030 | Enterovirus | Rhinovirus A55 | PV776129 |
| SBP-188 | Enterovirus | Rhinovirus A81 | PV776130 |
| SBP-119 | Enterovirus | Rhinovirus A15 | PV776131 |
| ICU-048 | Enterovirus | Rhinovirus A64 | PV776132 |
| SBA-018 | Enterovirus | Rhinovirus A | PV776133 |
| SBP-040 | Enterovirus | Rhinovirus A | PV776134 |
| SBP-121 | Enterovirus | Rhinovirus A | PV776135 |
| SBP-123 | Enterovirus | Rhinovirus A | PV776136 |
| SBP-169 | Enterovirus | Rhinovirus A | PV776137 |
| SBP-184 | Enterovirus | Rhinovirus A | PV776138 |
| SBP-135 | Enterovirus | Rhinovirus A32 | PV776139 |
| BTP-010 | Enterovirus | Rhinovirus C | PV776140 |
| ICU-012 | Enterovirus | Rhinovirus C | PV776141 |
| SAP-005 | Enterovirus | Rhinovirus C | PV776142 |
| SAP-007 | Enterovirus | Rhinovirus C | PV776143 |
| SBP-056 | Enterovirus | Rhinovirus C | PV776144 |
| SBP-050 | Enterovirus | Rhinovirus C | PV776145 |
| BTP-004 | Adenovirus | Human adenovirus 1 | PV776146 |
| SAP-007 | Adenovirus | Human adenovirus 1 | PV776147 |
| SAP-047 | Adenovirus | Human adenovirus 1 | PV776148 |
| SBP-035 | Adenovirus | Human adenovirus 5 | PV776149 |
| SBP-093 | Adenovirus | Human adenovirus 5 | PV776150 |
| SBP-155 | Adenovirus | Human adenovirus 5 | PV776151 |
| SBP-062 | Adenovirus | Human mastadenovirus C | PV776152 |
| SBP-066 | Adenovirus | Human mastadenovirus C | PV776153 |
| SBP-012 | Paramyxovirus | human respirovirus 3 | PV789149 |
| SBP-015 | Paramyxovirus | human respirovirus 3 | PV789150 |
| SBP-137 | Paramyxovirus | human respirovirus 3 | PV789151 |
| BTP001 | Coronavirus | SARS-CoV-2 | PV775393 |
| KPA007 | Coronavirus | SARS-CoV-2 | PV786829 |
| SBP067 | Coronavirus | SARS-CoV-2 | PV786830 |
| SBP152 | Coronavirus | SARS-CoV-2 | PV786831 |
| KPA058 | Coronavirus | Human coronavirus OC43 | PV789146 |
| SBA011 | Coronavirus | Human coronavirus OC43 | PV789148 |
| KPA062 | Coronavirus | Human coronavirus OC43 | PV789147 |
